# The effect of mobile phone addiction on sleep procrastination in adolescents: The longitudinal mediating role of physical activity behavior

**DOI:** 10.1371/journal.pone.0331340

**Published:** 2025-09-08

**Authors:** Wanbin Yu, Xielin Zhou, Bo Li, Jianjun Huang

**Affiliations:** 1 Department of Physical Education, Chengdu Sport University, Chengdu, Sichuan, China; 2 Department of Sports Training, Chengdu Sport University, Chengdu, Sichuan, China; 3 Department of Physical Education, Ningbo University, Ningbo, Zhejiang, China; University of Valencia: Universitat de Valencia, SPAIN

## Abstract

**Purpose:**

This study explores the impact of adolescent mobile phone addiction on sleep delay and analyzes the longitudinal mediating role of physical activity behavior. It provides a reference for cultivating good habits and healthy behaviors among adolescents.

**Methods:**

The study was based on the theory of planned behavior and used the Mobile Phone Addiction Scale (MPA), the Bedtime Procrastination Scale (BPS), and the Physical Activity Rating Scale to conduct a longitudinal follow-up survey of 376 healthy high school students in S Province. The survey was conducted three times at intervals of three months, and the data were processed using SPSS and AMOS.

**Results:**

(1) There is a positive correlation between mobile phone addiction and sleep delay (p < 0.01), and a negative correlation between mobile phone addiction, sleep delay, and physical activity (p < 0.01). (2) T1 mobile phone addiction significantly and negatively predicts T2 physical exercise behavior (β = −0.21, p < 0.001), T1 physical exercise behavior significantly and negatively predicts T2 sleep delay (β = −0.24, p < 0.001), and T1 mobile phone addiction significantly and positively predicts T2 sleep delay (β = 0.25, p < 0.001); T2 mobile phone addiction significantly and negatively predicts T 3 physical activity behavior (β = −0.27, p < 0.001), T2 physical activity behavior significantly negatively predicts T3 sleep delay (β = −0.37, p < 0.001), and T2 mobile phone addiction significantly positively predicts T3 sleep delay (β = 0.24, p < 0.001). (3) The direct effect of mobile phone addiction on physical exercise behavior is 0.08, with a confidence interval of [0.034, 0.153], indicating that the direct effect is established. The longitudinal mediating effect of sleep delay in mobile phone addiction and physical exercise behavior is 0.051, with a confidence interval of [0.02, 0.098], indicating that the longitudinal mediating effect is established.

**Conclusion:**

(1) There is a two-way correlation between adolescent mobile phone addiction, physical activity behavior, and sleep delay. (2) Adolescent mobile phone addiction can directly affect sleep delay across time and can also indirectly affect sleep delay across time through physical activity behavior.

## Introduction

The healthy physical and mental growth of adolescents is the cornerstone of the sustainable development of the country and society and is of great significance for realizing the strategy of strengthening the country through sports and building a healthy China. In 2016, the General Office of the State Council promulgated the “Opinions on Strengthening School Sports to Promote the Comprehensive Development of Students’ Physical and Mental Health,” emphasizing that “strengthening school sports is an important way to implement quality education and promote the comprehensive development of students. It is of great significance for promoting the modernization of education, building a healthy China and a strong country in human resources, and realizing the Chinese dream of the great rejuvenation of the Chinese nation [1]”. In 2023, the Ministry of Education and 17 other departments clearly defined the policy of “promoting mental health through the simultaneous development of the five key areas of education” in the “Special Action Plan for Comprehensively Strengthening and Improving the Mental Health of Young Students in the New Era (2023-2025) [2]”. This series of policy initiatives marks the continuous development of sports in schools in China and reflects the high degree of importance that the state and the education sector attach to the comprehensive development of students’ bodies and minds. However, with the rapid development of the social economy and profound changes in lifestyles, adolescents are facing many new challenges and pressures. One of these is the serious problem of sleep delay, which refers to an individual’s behavior of deliberately postponing the scheduled sleep time without a reasonable reason, thus reducing the actual sleep time [3]. This behavior is usually manifested in staying up late at night, and although the individual feels tired or knows that they need to rest, they still choose to continue engaging in other activities. According to survey data from China Youth Daily, it was further found that 65.3% of young people have sleep problems, and the reasons for their poor sleep quality and sleep delay are diverse, such as using electronic devices, watching videos, playing games, or engaging in social activities. Among them, electronic products have a relatively large impact on sleep delay [4].

The convenience of mobile phones makes it easier for individuals to develop addictive behaviors. If adolescents do not have external obstacles to prevent them from going to bed at a predetermined time, it will negatively affect their sleep quality and daily life [5,6]. Mobile phone addiction refers to an individual’s excessive use of mobile phones, dependence on mobile phones, and a pattern of behavior that affects daily life, work, study, and many other aspects [7]. The China Youth Daily public account reported on September 5, 2023, that “more than 97% of young people nationwide have sleep delay problems, and sleep quality has decreased by 35.8% [8].” This shows that the problem of sleep delay among young people in China is not optimistic at present. Research shows that sleep delay is a manifestation of low self-control and the result of a failure of self-regulation [9], and is susceptible to environmental factors, individual emotions, cognition, and behaviors (such as mobile phone addiction) [10]. Specifically, when individuals become addicted to screen-based activities, consuming a lot of time and energy, their investment in other activities decreases accordingly, exacerbating the phenomenon of procrastination in related activities [11]. Related studies have further found that the bedtime mobile phone usage behavior caused by mobile phone addiction is also gradually causing individuals to delay going to sleep [12]. The essence of mobile phone addiction lies in an individual’s over-reliance on mobile devices and lack of self-control. This lack of self-control makes it difficult for individuals to resist the urge to use their mobile phones, which in turn affects their ability to regulate themselves effectively [13]. When sleep delay becomes a habit, individuals have more time and opportunities to engage in other activities, and mobile phones, because of their entertainment and social nature, often become the preferred activity [14]. Based on this, this study proposes hypothesis H1: mobile phone addiction positively predicts sleep delay.

**Fig 1:**
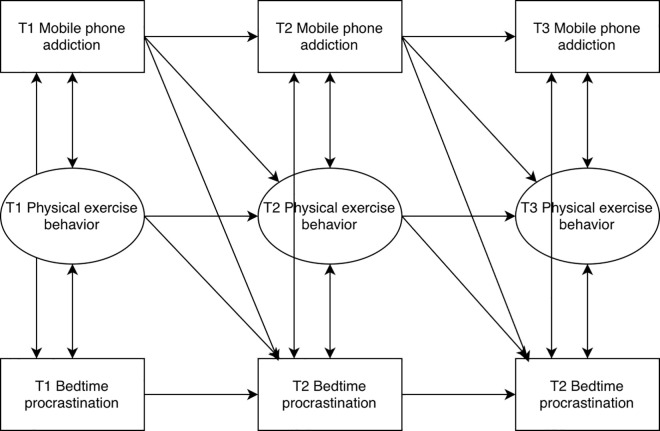
Longitudinal intermediate structure model diagram.

With the development of the Internet, mobile phones have become indispensable tools in the lives of modern people. The Statistical Report on the Development of China’s Internet Industry shows that by December 2023, the number of Internet users in China will reach 1.092 billion, and the Internet penetration rate will reach 77.5% [15]. This Fig 1 shows that people are becoming increasingly dependent on the Internet and mobile phones. While mobile phones bring convenience to people, they have also caused a series of adverse social phenomena, such as mobile phone addiction. Numerous studies have shown that mobile phone addiction harms adolescents’ behavior and health. From the behavioral level, mobile phone addiction weakens adolescents’ willingness to participate in physical activity, which further affects their physical and mental health, and due to prolonged screen behavior, they have difficulty in planning and carrying out regular physical activity, which triggers a series of chain reactions such as distraction and decline in physical function, resulting in the lower the motivation to invest in physical activity, and the more the habit of physical activity is difficult to develop [16]. From the theoretical level, according to the Theory of Planned Behavior, an individual’s behavior is subject to the joint effect of attitudinal tendencies, subjective norms, and behavioral perceived control, and when adolescents have negative attitudinal tendencies toward physical activity due to mobile phone addiction, their willingness to participate in physical activity will be reduced. At the same time, mobile phone addiction also affects their subjective norms to a certain extent, i.e., the expectations and pressures of people around them on physical activity have a weaker influence on them. In addition, mobile phone addicts often lack effective control over their behavior and find it difficult to resist the temptation of the mobile phone, and thus, there is also a bias in the perceived control of their behavior, which makes it difficult for them to put physical exercise into practice. In summary, combined with actual behavioral performance and theoretical analysis, mobile phone addiction can negatively affect physical activity behavior. Physical exercise, as an active lifestyle, promotes healthy physical and mental development of adolescents and changes behavioral habits and behaviors. The Theory of Planned Behavior states that an individual’s behavior is controlled by a combination of attitudinal dispositions, subjective norms, and perceived control of behavior. Based on the Theory of Planned Behavior, a study further found that regular physical activity strengthens an individual’s time management ability, possesses higher self-control, and rationally plans daily activities [17]. This improvement in time management and self-control enables adolescents to better arrange their own schedules and reduce sleep delay behaviors caused by addiction to mobile phones or other electronic devices. Based on this, this study proposes hypothesis H2: Physical exercise behavior plays a longitudinal mediating role in the impact of mobile phone addiction on bedtime delay.

## Materials and methods

### Participants

This study used stratified sampling method to ensure that the sample can fully represent the situation of high schools of different schooling nature in Province S. According to the differentiation of the nature of public and private schooling, high schools in two cities of similar educational level in Province S were selected, among which two private high schools and two public high schools were stratified, and in each high school, for each grade, simple random sampling method was used, and one administrative class was selected as the most sub-sampling unit in each high school, and A longitudinal follow-up survey was conducted for 3 months. Before distributing the questionnaires, the class teachers were trained on the survey process and key points. The three surveys were conducted in a consistent manner, all using an online questionnaire. Before each survey, the class teacher explained the purpose and use of the study to the subjects, emphasizing the voluntary nature of participation, and allowing subjects to abandon the test midway if they wished. Subjects were informed of how the survey data would be stored and the confidentiality pledge. Before administering the questionnaire, the minimum sample size was calculated by using the G*Power program, setting α = 0.05, β = 0.80, Effect Size = 0.15, and obtaining the minimum sample size required for the study as 138. A total of 432 questionnaires were distributed three times. The first set of data was collected in September 2024 (T1), with 419 questionnaires returned. The second set of data was collected in October 2024 (T2), with 411 questionnaires returned. The third set of data was collected in November 2024 (T3), with 406 questionnaires returned (01/09/2024–05/11/2024). The subjects who chose to complete the questionnaire in all three surveys were selected as the final valid samples. Invalid questionnaires were collected, and invalid questionnaires were collected and discarded, with 376 valid subjects in the end, an effective rate of 87.04%. Among them, there were 180 male students, accounting for 47.9%, and 196 female students, accounting for 52.1%, with an average age of 16.09 ± 1.18 years old.

### Ethics statement

All methods were carried out according to relevant guidelines and regulations. The studies involving human participants were reviewed and approved by the Chengdu Sport University ethics committee (Ethical Approval Number: 202493). The participants provided their written informed consent to participate in this study.

### Methods

#### Mobile Phone Addiction Scale.

A 5-point Likert scale was used, with each item having 5 levels (1 = strongly disagree; 2 = disagree; 3 = undecided; 4 = agree; 5 = strongly agree). Regarding the Mobile Phone Addiction Scale compiled by previous researchers [18], there are 22 items in total, including 6 factors: withdrawal behavior, salient behavior, social pacification, negative impact, app use, and app updates. The higher the score, the more severe the mobile phone addiction. Validated factor analysis yielded good fit indicators for the 6-factor conceptualization structure, $\chi^{\text{2}}/df$=1.57, CFI = 0.92, IFI = 0.93, RMSEA = 0.05, proving that the scale has good reliability and validity. The Cronbach alpha coefficients for the three tested time points in this study through SPSS were 0.964, 0.950 and 0.952, respectively, while a validation factor analysis was conducted using AMOS in order to test the validity of the scale in the sample of the current study, and the results showed that: $\chi^{\text{2}}/df$=1.103, GFI = 0.947, CFI = 0.996, IFI = 0.996, TLI = 0.996, RMSEA = 0.017 (Test1); $\chi^{\text{2}}/df$=1.490, GFI = 0.931, CFI = 0.975, IFI = 0.975, TLI = 0.973, RMSEA = 0.036 (Test2); $\chi^{\text{2}}/df$=1.374, GFI = 0.934, CFI = 0.982, IFI = 0.982, TLI = 0.980, RMSEA = 0.032 (Test3), this indicates that the scale has good reliability.

#### Bedtime Procrastination Scale.

According to the Chinese version of the Bedtime Procrastination Scale compiled by Kroese and revised by Zhang Lu and other scholars [19], there are 9 items in total, using a 5-point Likert scale (1 = never; 2 = sometimes; 3 = sometimes; 4 = often; 5 = always). Four of the entries were reverse scoring questions, with higher scores on the scale indicating a more severe tendency to sleep procrastination, and in their study the Cronbach α coefficient was 0.835, and the retest reliability was 0.72, which possessed a high degree of validity scale validity. In this study, the Cronbach α coefficients at the T1, T2, and T3 time points were 0.918, 0.883, and 0.897 respectively. The results of the confirmatory factor analysis showed that the results of the confirmatory factor analysis at the three time points were as follows: $\chi^{\text{2}}/df$=1.184, GFI = 0.982, CFI = 0.997, IFI = 0.997, TLI = 0.996, RMSEA = 0.022 (Test1); $\chi^{\text{2}}/df$=1.134, GFI = 0.983, CFI = 0.997, IFI = 0.997, TLI = 0.996, RMSEA = 0.019 (Test2); $\chi^{\text{2}}/df$=1.418, GFI = 0.978, CFI = 0.992, IFI = 0.992, TLI = 0.990, RMSEA = 0.033 (Test3), this indicates that the scale has good reliability.

#### Physical Activity Behavior Scale.

The Physical Activity Rating Scale compiled by Liang Deqing was used to measure physical activity intensity, duration, and frequency. A 5-point Likert scale was used, and the product of the scores for the three dimensions was calculated as the total physical activity score, with light, moderate, and vigorous exercise intensity used to classify the activity level [20]. The Cronbach’s alpha coefficients at time points T1, T2, and T3 were 0.828, 0.688, and 0.730 respectively. The results of the exploratory factor analysis were as follows: KMO coefficient of 0.708, p < 0.001 (Test1) in the Bartlett sphericity test; KMO coefficient of 0.664, p < 0.001 (Test2) in the Bartlett sphericity test; and KMO coefficient of 0.683, p < 0.001 (Test3) in the Bartlett sphericity test, indicating that the scale has good reliability.

### Data analysis

The collected data were processed and analyzed using SPSS 27.0 and AMOS 26.0 for common method bias testing, descriptive statistics (mean, standard deviation, etc. for each variable), correlation analysis, independent samples t-tests, and ANOVA with validation factor analysis through SPSS. AMOS was utilized for model construction and examination between variables, in the AMOS graphical interface, the measurement model was constructed based on theory, the estimation of model parameters was performed using the method of great likelihood (ML), and the metrics for evaluating the model were adopted $\chi^{2}/df$<5, CFI > 0.90, IFI > 0.90, GFI > 0.90, and RMSEA<0.08. and test the autoregressive coefficients and longitudinal mediation of the model.

## Results

### Common methodology bias test

The Harman single-factor test was used to test for common method bias [21]. The results showed that three factors with three characteristic roots greater than 1 were extracted at each of the three time points (T1, T2, and T3). The first factor explained 39.96%, 36.23%, and 37.36% of the total variance, respectively, all of which were less than the critical value of 40% [22], indicating that there was no serious common method bias in any of the three measurements.

### Descriptive statistics and correlation analysis

Using SPSS 27.0, an independent sample T-test for gender and a one-way analysis of variance by school stage were performed for each of the three variables measured three times. In the Levene’s test of equality of variance by gender, none of the variables were significant (p > 0.05), the null hypothesis was accepted, and the data was assumed to be homoscedastic (Table 1). The T test for the equality of means by gender showed that there were no significant gender differences in the three tests of mobile phone addiction, sleep delay and physical exercise behavior (p > 0.05). A one-way ANOVA was performed on school stage and age (Table 2), and the results showed that there were also no significant differences in the three tests of mobile phone addiction, sleep delay and physical exercise behavior by school stage and age (p > 0.05).

**Table 1:** Independent samples t-test for gender in T1, T2 and T3.

			Levene’s equality of variance test		Mean value equivalence t test	
Group variable			F	p	t	p
Gender	T1 Mobile phone addiction	Hypothesis of equal variance	1.739	0.188	−0.468	0.64
	T1 bedtime procrastination	Hypothesis of equal variance	0.463	0.497	−0.43	0.667
	T1 physical activity behavior	Hypothesis of equal variance	0.049	0.825	−0.115	0.909
	T2 Mobile phone addiction	Hypothesis of equal variance	1.252	0.264	−0.09	0.928
	T2 bedtime procrastination	Hypothesis of equal variance	1.093	0.297	−0.631	0.529
	T2 physical activity behavior	Hypothesis of equal variance	0.016	0.901	−0.966	0.335
	T3Mobile phone addiction	Hypothesis of equal variance	0.426	0.514	−1.835	0.067
	T3 bedtime procrastination	Hypothesis of equal variance	0.002	0.963	0.091	0.927
	T3 physical activity behavior	Hypothesis of equal variance	0.396	0.529	1.217	0.224

**Table 2:** ANOVA for grade and age for T1, T2 and T3.

Group variable	Dependent variable	Root mean square	F	p
Grade	T1 Mobile phone addiction	0.659	0.906	0.460
	T1 bedtime procrastination	1.189	1.541	0.190
	T1 physical activity behavior	1.624	1.344	0.110
	T2 Mobile phone addiction	0.197	0.306	0.874
	T2 bedtime procrastination	0.035	0.051	0.995
	T2 physical activity behavior	0.96	1.092	0.360
	T3Mobile phone addiction	0.27	0.404	0.806
	T3 bedtime procrastination	0.892	1.118	0.347
	T3 physical activity behavior	1.316	1.311	0.265
Age	T1Mobile phone addiction	0.714	0.983	0.416
	T1 bedtime procrastination	0.272	0.349	0.845
	T1 physical activity behavior	0.435	0.389	0.816
	T2 Mobile phone addiction	0.32	0.497	0.738
	T2 bedtime procrastination	0.904	1.32	0.262
	T2 physical activity behavior	0.177	0.199	0.939
	T3 Mobile phone addiction	0.661	0.993	0.411
	T3 bedtime procrastination	0.338	0.421	0.793
	T3 physical activity behavior	1.804	1.806	0.127

The results of the correlation analysis of the three variables of adolescents’ mobile phone addiction, sleep procrastination and physical activity behavior are shown (Table 3): a significant positive correlation (p < 0.01) was found between T1 mobile phone addiction (r = 0.439), T3 mobile phone addiction (r = 0.390), T2 mobile phone addiction (r = 0.511), T1 sleep procrastination (r = 0.412) and T2 sleep procrastination (r = 0.300) and T3 sleep procrastination (r = 0.346), T2 sleep procrastination showed a significant positive correlation with T3 sleep procrastination (r = 0.412) (p < 0.01), and T1 physical activity behavior showed a significant positive correlation with T2 physical activity behavior (r = 0.270), T3 physical activity behavior (r = 0.269), and T2 physical activity behavior with T3 physical activity behavior (r = 0.361) showed a significant positive correlation (p < 0.01). Significant correlations (p < 0.01) were presented between mobile phone addiction, sleep procrastination and physical exercise behavior at the three time points, with T1 mobile phone addiction showing a significant positive correlation (p < 0.01) with T2 sleep procrastination (r = 0.360) and T3 sleep procrastination (r = 0.375), and T1 mobile phone addiction showing a significant positive correlation (p < 0.01) with T2 physical exercise behavior (r = −0.231) and T3 physical exercise behavior (r = −0.290) showed a significant negative correlation (p < 0.01), which satisfied the preliminary conditions for cross-lag model construction.

**Table 3:** Mean, standard deviation, and correlation analysis.

	X1	Y1	Z1	X2	Y2	Z2	X3	Y3	Z3
T1 Mobile phone addiction	1								
T1 physical activity behavior	−.319**	1							
T1 bedtime procrastination	.277**	−.432**	1						
T2 Mobile phone addiction	.439**	−.305**	.379**	1					
T2 physical activity behavior	−.231**	.270**	−.210**	−.303**	1				
T2 bedtime procrastination	.360**	−.334**	.300**	.433**	−.255**	1			
T3 Mobile phone addiction	.390**	−.350**	.362**	.511**	−.305**	.422**	1		
T3 physical activity behavior	−.290**	.269**	−.286**	−.339**	.361**	−.318**	−.333**	1	
T3 bedtime procrastination	.375**	−.358**	.346**	.433**	−.387**	.412**	.423**	−.360**	1
M	3.09	3.12	3.10	3.10	3.21	3.10	3.04	3.14	3.06
SD	0.85	1.05	0.88	0.80	0.94	0.83	0.82	1.00	0.89

“*” indicates p < 0.05, “**” indicates p < 0.01 (due to space constraints in the table, X1 = T1 mobile phone addiction, Y1 = T1 physical exercise behavior.

### Longitudinal mediation test and analysis of physical activity behavior

The structural equation model established through AMOS 26.0 uses a latent variable model for the physical activity behavior dimension due to the high correlation coefficients between the intensity, duration, and frequency of physical activity. The fit indices using the maximum likelihood method indicate that: $\chi^{\text{2}}/df$=2.971, GFI = 0.927, CFI = 0.905, IFI = 0.906, TLI = 0.877. RMSEA = 0.097,Indicates that the model fits the data well (Table 4).

**Table 4:** Indicators of model fit.

Model Name	$\chi^{2}/df$	GFI	CFI	IFI	TLI	RMSEA
M2	2.971	0.927	0.905	0.906	0.877	0.073

**Fig 2:**
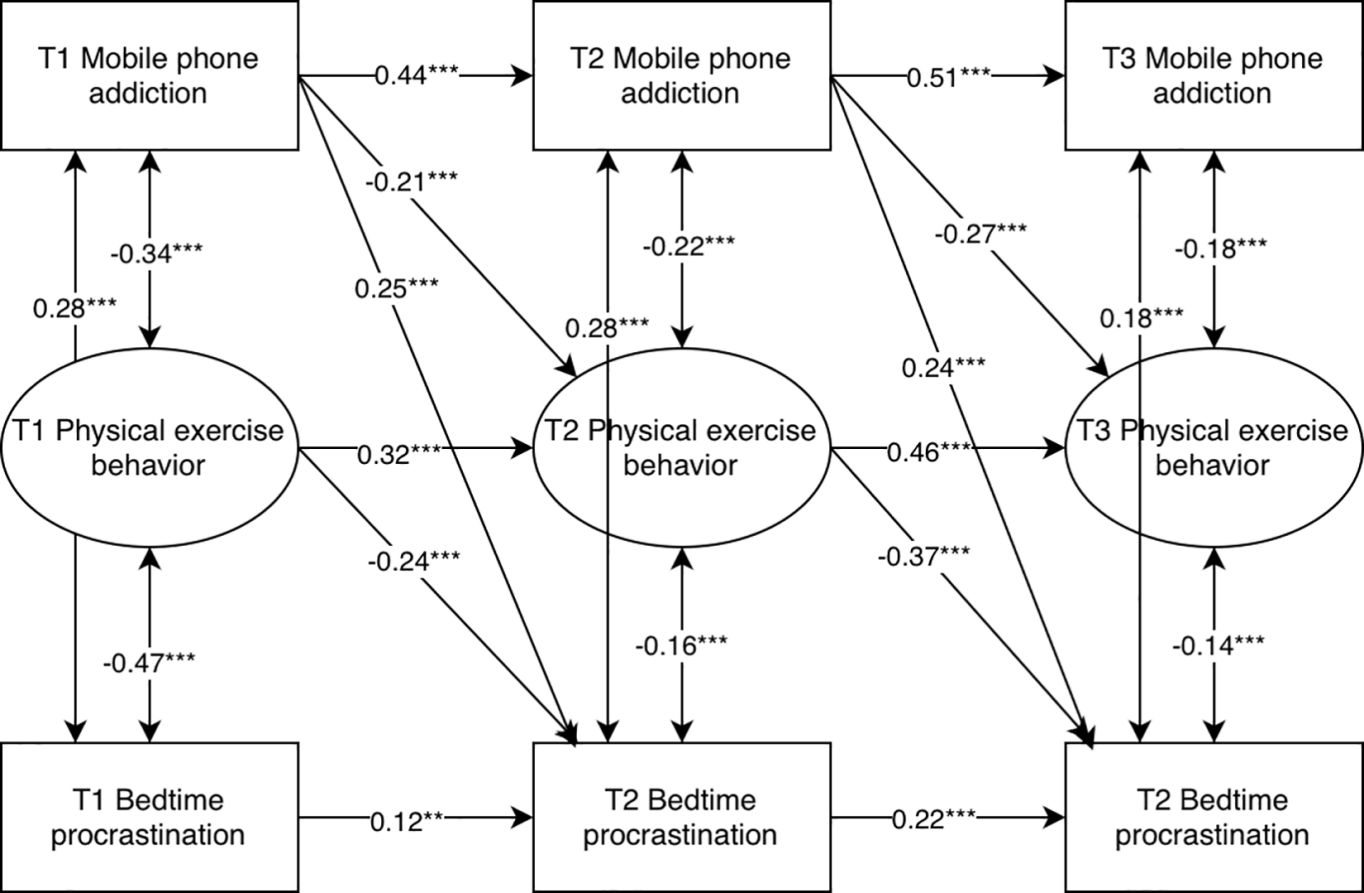
Longitudinal mediation analysis of physical exercise behavior.

The results showed that T1 mobile phone addiction significantly and negatively predicted T2 physical activity behavior (β = −0.21, p < 0.001), T1 physical activity behavior significantly and negatively predicted T2 sleep delay (β = −0.24, p < 0.001), and T1 mobile phone addiction significantly and positively predicted T2 sleep delay (β = 0.25, p < 0.001); T2 mobile phone addiction significantly and negatively predicted T 3 physical exercise behavior (β = −0.27, p < 0.001), T2 physical exercise behavior significantly negatively predicts T3 sleep delay (β = −0.37, p < 0.001), and T2 mobile phone addiction significantly positively predicts T3 sleep delay (β = 0.24, p < 0.001) (Fig 2). Combining the path coefficients of the longitudinal mediation model, the path from T1 mobile phone addiction to T2 physical exercise behavior is a1, and the path from T1 physical exercise behavior to T2 sleep delay is b1; the path from T2 mobile phone addiction to T3 physical exercise behavior is a2, and the path from T2 physical exercise behavior to T3 sleep delay is b2. The bootstrap method was used for testing, and the confidence interval was set at 95%. The direct effect value of mobile phone addiction on physical exercise behavior was 0.08, CI=[0.034,0.153], indicating that the direct effect is established; the longitudinal mediating effect of sleep delay in mobile phone addiction and physical exercise behavior is 0.051, CI=[0.02,0.098], indicating that the longitudinal mediating effect is established.

## Discussion

### The impact of adolescent mobile phone addiction on sleep delay

This study used a longitudinal mediation model to confirm the longitudinal mediating effect of physical exercise behavior in the influence of adolescent mobile phone addiction on sleep delay. At the same time, the direct effect of adolescent mobile phone addiction on sleep delay was significant. Adolescent mobile phone addiction can positively predict sleep delay, which is consistent with the results of previous studies [23]. Smartphones play an increasingly important role in modern life, providing many conveniences and greatly facilitating communication and exchanges between individuals. However, with the popularity of smartphones, adolescents’ mobile phone usage time is increasing, and their dependence is gradually increasing. Many adolescents are addicted to the Internet and short videos, and they have difficulty controlling themselves, which has a serious impact on their physical and mental health and has led to sleep delay behavior problems [24]. Specifically, adolescents’ lack of self-control is an important factor leading to sleep delay [17], and mobile phone addiction is a typical manifestation of adolescents’ lack of self-control [25]. In daily life, excessive screen time behavior leads to unreasonable allocation of time, which delays sleep time [26]. The theory of self-control further suggests that when adolescents develop mobile phone addiction problems, they need more mental resources to control the time of mobile phone use and self-regulate [27]. Therefore, the higher the degree of mobile phone addiction, the fewer mental resources adolescents can obtain, the lower their control, and the more difficult it is for them to rest on time. Therefore, mobile phone addiction can positively affect sleep delay over time, further confirming the long-term negative impact of mobile phone addiction on adolescent health.

### Longitudinal mediation of physical activity behavior

The study found that physical activity behavior plays a longitudinal mediating role in the impact of adolescent mobile phone addiction on sleep delay. When adolescents have a mobile phone addiction, they tend to devote more time and energy to mobile phone use, which leads to their leisure time being occupied by screen-based behaviors, thereby reducing physical exercise behavior [28]. This pattern of behavior not only harms the physical and mental health of adolescents, but also causes many inconveniences in their studies and lives. The Internet displacement hypothesis further elaborates on this phenomenon. When individuals spend too much time on mobile phone use, they devote less energy to other activities [29]. Previous studies have also confirmed that mobile phone addiction can lead to negative emotions in adolescents, causing them to become physically inactive and doubt their ability to exercise [30]. This dual mediation pathway suggests that physical activity mediates smartphone addiction’s impact on sleep procrastination through both energy expenditure mechanisms and habitual behavior modification. The Theory of Planned Behavior suggests that an individual’s intention to act is an antecedent to engaging in an activity, and that the intention itself is synergistically influenced by attitudes, subjective norms, and perceived behavioral control [31]. Specifically, individuals who have positive attitudes toward physical activity, feel positive support from the social environment, and have high confidence in their ability to perform physical activity are significantly more likely to engage in physical activity participation [32]. It has been established that regular physical activity enhances an individual’s time management skills and self-control [33], and that these abilities help to improve sleep habits. Therefore, the more motivated adolescents are to engage in physical activity, the less sleep procrastination is likely to occur. Based on the Internet displacement hypothesis and the theory of planned behavior, this study proposes the model of “restricted behavioral resources lead to weakened decision-making ability”. On the one hand, mobile phone addiction consumes adolescents’ behavioral resources excessively, which directly “displaces” the opportunity and intention to participate in physical activity, leading to a decrease in physical activity behavior. On the one hand, mobile phone addiction consumes excessive behavioral resources of adolescents, directly “displacing” the opportunity and intention to participate in physical activity, leading to a decrease in physical activity behavior. On the other hand, the reduction of physical activity weakens the key core competencies of individuals in sleep management (positive attitude, perceived behavioral control), and the weakening of this decision-making and control ability makes adolescents lack sufficient psychological resources to carry out the plan, which ultimately manifests itself in sleep procrastination. Therefore, in secondary school education, the occurrence of sleep procrastination should be effectively reduced by enhancing individuals’ positive attitudes, positive subjective norms, and perceptual behavioral control, and at the same time, adolescents should be guided and encouraged to participate in physical exercise to help them develop healthy living habits and promote their overall development.

Although the statistical results show that there is no significant gender difference in various variables, male and female adolescents may have different preferences in terms of the intensity and form of exercise. Therefore, when formulating strategies to prevent excessive mobile phone use and personalized physical exercise plans in the future, the potential differences between genders in various variables based on the concept of individual differences and teaching students according to their aptitude still deserve careful consideration.

### Strengths and limitations of the study

This study innovatively combines the widespread phenomenon of mobile phone addiction, the increasingly prominent problem of sleep delays, and the health-promoting behavior of physical activity among adolescents, in order to explore the intrinsic connection between the three, and precisely focus on the important issues of physical and mental health development of contemporary adolescents. In terms of research design, this study used physical activity as a mediator variable and utilized a longitudinal tracking design, which effectively solved the problems of transient interference and poor stability that existed in previous cross-sectional studies. From the perspective of practical application, this study can help educators, parents, and other groups to better understand the potential threat of adolescent mobile phone addiction to sleep health and the buffering effect of physical activity. Based on this, relevant personnel can formulate targeted interventions, such as guiding adolescents to rationally plan the use of mobile phones and motivating them to actively engage in physical activity, which is important for improving adolescents’ sleep delays and enhancing their overall health. However, there are still some shortcomings in this study, and future research can be improved in the following aspects:

(1) In terms of school stratification, this study was only based on the nature of the school (public, private). Future studies should further optimize the sample selection, and in addition to considering geographical factors (rural, urban), should also cover adolescents of different ages, and conduct comparative analyses between junior high school students and senior high school students, in order to enhance the comprehensiveness and universality of the study’s conclusions.(2) Although this study controlled for a number of basic demographic variables, there may have been other uncontrolled confounding factors. For example, factors such as family environment, school climate, peer relationships, and adolescents’ personality traits may have an impact on mobile phone addiction, physical activity behaviors, and sleep procrastination. Future studies may further consider covariates such as gender and school type for enhanced analysis as appropriate. (3) Given the focus on the longitudinal mediating role of physical activity behavior between mobile phone addiction and sleep procrastination, this study employed a traditional cross-lagged panel model (CLPM), which has some limitations. Future studies can introduce random intercepts and use the random intercept cross-lagged model (RI-CLPM) to capture stable traits of individuals (e.g., baseline of individual sleep habits), to analyze the dynamic mechanisms in more depth, and to provide more detailed and precise theoretical support and empirical evidence for research in this field.

## Conclusion

This study explored the impact of adolescent mobile phone addiction on sleep delay through longitudinal tracking surveys and longitudinal mediation model design and analysis and explored the longitudinal mechanism of physical exercise behavior. The conclusions are of practical significance for improving the problems of adolescent mobile phone addiction and sleep quality. However, due to the length, of follow-up studies, scholars can expand the sample size and further explore the impact of the demographic variable of profession by investigating adolescents in the region. At the same time, in addition to mobile phone addiction and physical exercise behavior, it is still necessary to pay attention to and consider the possible role and impact of other factors on adolescents’ sleep delay, and other variables can also be considered for research.

## Supporting information

S1 File Data. (XLSX)
